# The Role of *N*6-Methyladenosine (m6A) RNA Modification in the Pathogenesis of Parkinson’s Disease

**DOI:** 10.3390/biom15050617

**Published:** 2025-04-23

**Authors:** Yulu Wang, Tianyuan Zhao, Chunsen Yuan, Xuechai Chen

**Affiliations:** College of Chemistry and Life Science, Beijing University of Technology, 100 Pingleyuan, Chaoyang District, Beijing 100124, China; wangyulu@emails.bjut.edu.cn (Y.W.); zhaoty719@emails.bjut.edu.cn (T.Z.); ycs18910519729@emails.bjut.edu.cn (C.Y.)

**Keywords:** m6A RNA modification, Parkinson’s disease, clinical research, dopamine, genetic genes

## Abstract

Parkinson’s disease (PD) is a neurodegenerative disease with a high prevalence among the middle-aged and elderly population. The pathogenesis of PD is closely linked to the misfolding and aggregation of α-synuclein, which contributes to the formation of Lewy bodies. These processes are associated with the degeneration of dopaminergic neurons, a key neuropathological change that underlies the motor symptoms of PD. In addition, genetic susceptibility, mitochondrial dysfunction, oxidative stress and neuroinflammation are involved in the progress of the disease. Previous studies indicated that the dysregulation of epigenetic modifications, including DNA methylation and histone acetylation, may be the key pathophysiological factors in PD. *N*6-methyladenosine (m6A) is a dynamically reversible modification in eukaryotes RNA, and could regulate mRNA degradation, stability, maturation, and translation. Recently, clinical research has shown that the global m6A level is significantly reduced in PD patients as well as the expression changes in m6A-associated proteins. Moreover, the dysregulation of m6A modification was shown to impact dopamine metabolism and damage dopaminergic neurons, indicating that m6A RNA modification may play a critical role in the pathogenesis of PD. In this review, we summarize recent clinical studies on m6A RNA modification in PD patients and discuss the regulatory role of m6A modification in dopamine metabolism and dopaminergic neurons death. Furthermore, based on the different m6A modification databases and prediction websites, we analyzed the potential m6A modification sites on the mRNA of key PD pathogenic genes (*SNCA*, *PRKN*, *PINK1*, and *LRRK2*) for the first time, aiming to offer new gene targets and perspectives understanding the pathogenesis of PD.

## 1. Introduction

In recent years, chemical modifications of RNA, collectively called the “epitranscriptome”, have been found to regulate various aspects of RNA function and metabolism, which have diverse impacts on gene expression and may provide novel biomarkers and innovative targets for cancer, cardiovascular disease and neurodegenerative disease. *N*6-methyladenosine (m6A), the methylation of the *N*6 site of adenosine, is the most common and widely studied methylation modification, which appears on mRNA, lncRNA and other RNA. m6A RNA modification usually appears in the position of adenine within DRACH motif (where D = A, G or U; R = A or G; H = A, C or U) [1]. Its function is involved in the process of generation, removal and recognition and regulated splicing, stability, translation, and intracellular localization of transcripts [2].

m6A modification is dynamically installed by a conserved group of methyltransferases termed “writers”. The core catalytic unit comprises the m6A methyltransferase-like (METTL) METTL3-METTL14 heterodimer, where METTL3 transfers methyl groups from S-adenosylmethionine to adenosine residues in DRACH motifs, while METTL14 stabilizes RNA binding and enhances catalytic efficiency [3]. This complex is regulated by the Wilms tumor 1-associated protein (WTAP), which directs its localization to nuclear speckles and recruits auxiliary factors like vir-like m6A methyltransferase-associated (VIRMA), zinc finger CCCH-type containing13 (ZC3H13), and RNA-binding motif protein 15/15B (RBM15/15B) to modulate substrate specificity, such as targeting 3′UTRs or noncoding RNAs. METTL16 methylates snRNA and acts as a SAM sensor, effectively linking m6A deposition to metabolic homeostasis [4]. METTL5 is mainly responsible for catalyzing the m6A modification of 18S rRNA [5]. The dysregulation of “writers”, such as the deficiency of METTL3 or METTL14, inhibits the development of neurons [6], underscoring their pathological significance in neurodegenerative diseases [7]. m6A modifications can be erased by demethylases (known as erasers); the two known demethylases are the fat mass and obesity-associated proteins (FTO) and AlkB homologue 5 protein (ALKBH5) [8,9]. The identification of these demethylases highlights the reversibility of m6A modification. In the nucleus, FTO facilitates the demethylation of approximately 5% to 10% of mRNA by preferentially targeting m6A in specific RNA contexts, such as near splice sites and within coding sequences, impacting mRNA splicing [10]. ALKBH5, localized in nuclear speckles, targets m6A on nascent transcripts and lncRNAs, facilitating mRNA export by resolving RNA-protein interactions [11].

The protein that recognizes and binds to m6A-modified RNA is the reader of m6A modification, which exhibits two modes of m6A-dependent RNA binding: direct and indirect. Direct binders include YTH domain-containing proteins (YTHDF1-3 and YTHDC1-2). YTHDF1 enhances translation by recruiting initiation factors, such as eukaryotic translation initiation factor 3 (eIF3), to m6A-marked mRNAs, whereas YTHDF2 promotes mRNA decay by directing transcripts to processing bodies through interactions with the CCR4-NOT deadenylase complex. YTHDF3 synergizes with both YTHDF1 and YTHDF2 to regulate translation and decay [12]. YTHDC1 facilitates mRNA splicing by binding m6A sites near exon-intron junctions [13]. Furthermore, YTHDC2 enriches in germ cells and enhances translation efficiency of m6A-modified transcripts by resolving secondary RNA structures [14]. Non-YTH readers, such as IGF2BPs (IGF2BP1-3) and heterogeneous nuclear ribonucleoprotein C (HNRNPs), have distinct roles in mRNA stability [15]. IGF2BPs protect oncogenic mRNAs from YTHDF2-mediated decay, while HNRNPs utilize an m6A-switch mechanism, where m6A-induced RNA unfolding exposes buried RNA-binding motifs, resulting in HNRNPC binding and promoting exon inclusion.

m6A RNA modification is particularly abundant in the brain and is involved in embryonic stem cell differentiation, brain development, and neurodevelopmental disorders [16]. Existing studies have confirmed that a high methylation environment in the brain can significantly enhance memory formation in the medial prefrontal cortex and the dorsal hippocampus [17], axon regeneration [18], and neuronal differentiation [19,20]. The prominent role of m6A modification in the central nervous system suggests that dysregulation of m6A may be associated with neurological diseases. An increasing number of studies report that changes in m6A RNA modification levels are involved in the pathogenesis of Alzheimer’s disease [21], Parkinson’s disease (PD) [22], depression [23,24], and epilepsy [25].

PD is one of the most common neurodegenerative disease characterized by tremors and bradykinesia, predominantly affecting the elderly and males [26]. The main neuropathological feature of PD is the damage to dopamine neurons in the substantia nigra (SN) [27], which leads to dopamine depletion in the striatum and ultimately results in clinical symptoms [28]. Related studies have provided evidence that progressive degeneration of vulnerable dopaminergic neurons may be caused by a combination of cellular disturbances due to misfolding and aggregation of α-synuclein (α-Syn) [29], disruption of the autophagy-lysosome system [30], and dysregulation of calcium homeostasis [31]. However, the specific pathogenesis of PD has not been fully elucidated. Chen et al. initially found that modification of m6A by FTO can lead to the death of dopaminergic cells and may be essential in the pathogenesis of PD [22]. Subsequently, a growing number of studies have demonstrated that changes in the level of m6A RNA modification and in the expression of m6A-related proteins can contribute to the development of PD.

Exploring the impact of m6A modification on the pathogenesis of PD provides new insights into understanding the mechanisms underlying PD and is significant for identifying new biomarkers for the disease. In this review, we summarize clinical studies on the m6A modification levels in PD patients, discussing the effects of m6A on dopaminergic cells from various perspectives and making reasonable speculations about other possible pathways. Additionally, we outline the m6A modification sites of specific pathogenic genes associated with PD. These efforts are crucial for deepening our understanding of the pathogenesis of PD and for discovering new diagnostic markers and therapeutic directions.

## 2. Clinical Studies of m6A RNA Modification in PD

PD common motor symptoms include bradykinesia, resting tremor, rigidity, and postural instability [32], and these symptoms always serve as primary focal points during clinical diagnosis. However, early stage PD may present with inconspicuous clinical features, leading to a certain rate of misdiagnosis [33]. At least 50% of SN neurons are lost [34] when the first motor and cognitive symptoms appear, which poses substantial challenges for disease mitigation and treatment. Therefore, understanding the biological characteristics within PD patients is crucial for early prediction. Various biomarkers for an early diagnosis of PD include imaging, cerebrospinal fluid, oxidative stress and neuroinflammation [35]. Multiple proteins are considered as potential biochemical markers for PD, such as glial fibrillary acidic protein, DJ-1 and neurofilament light chain protein in cerebrospinal fluid [35]. The research on PD necessitates the identification of a unique biomarker that can distinguish PD from other diseases with higher sensitivity and specificity. Recently, significant changes in m6A RNA modification levels and m6A regulators in clinical studies of PD have drawn attention.

**Figure 1:**
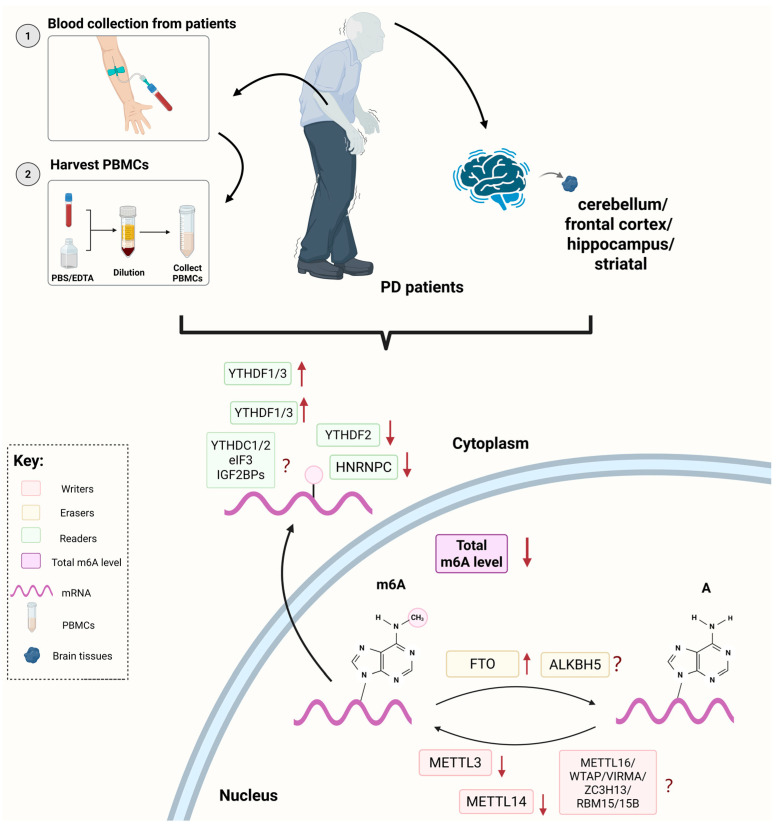
The changes in global m6A level and its regulatory proteins expression in the PD patients. The total m6A level in the PD patients was decreased compared to the healthy control. The m6A modification is regulated by “writers”, “erasers” and “readers”. The arrows indicate the expression changes in the m6A regulatory proteins in PBMCs extracted from the PD patients’ blood or in different regions of brain tissues. The question marks represent that the regulatory proteins that have not been studied or the expression changes that are inconsistent in different research. Created with BioRender.com.

The changes in global m6A level and its regulatory proteins expression in the PD patients are summarized in Figure 1. Currently, the clinical samples are derived from peripheral blood mononuclear cells (PBMCs) extracted from the blood samples and tissue samples from different regions of the brains of the PD patients. Xiao et al. found that, compared to the healthy controls, the overall m6A modification levels in the PBMCs of the PD patients were reduced [36]. Martinez et al. used microscopy and machine learning for cellular profiling and discovered that the m6A modification levels in the cerebellum, frontal cortex, and hippocampus of the PD patients’ brain tissues performed significantly lower [37]. In addition, the expression level of m6A regulatory protein has emerged as a focal point in the clinical study of PD. In the aforementioned study, Xiao et al. found that the protein expression levels of METTL3, METTL14 and YTHDF2 in PBMCs were significantly reduced, with METTL14 being the primary factor influencing the decrease in m6A modification levels. The Spearman correlation analysis indicated a moderate negative correlation between the level of METTL14 and α-Syn content in the plasma of the PD patients, suggesting that the combination of these factors has excellent diagnostic potential for PD. However, the changes in YTHDF1/3 in the three brain regions mentioned above were inconsistent. Geng et al. analyzed the differential expression of m6A modification-related genes in the striatal tissues of six PD patients and six healthy individuals from the GSE54282 dataset [38], revealing that the expression of FTO and YTHDF3 was significantly elevated. Combining these results, it is clear that certain representative methyltransferases typically show decreased expression in PD patients, while the expression of demethylases were elevated. Given the current research progress, it can be speculated that m6A regulatory factors may ultimately influence the pathogenesis or phenotype of PD by affecting the methylation levels of PD-related proteins. However, existing findings require further clinical validation, and the changes in other m6A regulators in PD also need to be revealed.

At the genetic level, Qiu et al. combined the genome-wide association study with the expression quantitative trait loci to identify five m6A-related single nucleotide polymorphisms (m6A-SNPs) significantly associated with PD: rs75072999 in G-associated kinase (GAK); rs1033500 in C6orf10; and rs1378602, rs4924839, and rs8071834 in ALKBH5 [39]. SNPs are single nucleotide variations that exist among individuals at specific positions in the genome. Changes in the RNA sequences of m6A modification targets can affect mRNA stability, potentially leading to the onset of neurodegenerative diseases [40]. Combining the intrinsic functions of proteins is conducive to the investigation of the potential roles of m6A-SNPs and their associated genes in the risk of PD. The reduced GAK function may enhance α-syn mediated toxicity in PD [41]. The m6A-SNP rs1564282 in GAK have been demonstrated to be associated with the risk of PD [42]. The role of rs75072999 in the risk of PD still remains to be explored. The specific function of ALKBH5 in PD remains unclear. It is primarily expressed in the nucleus of neurons and cell lines in mice, and is involved in the development of the nervous system [43]. Identifying the m6A-SNPs of ALKBH5 provides support for exploring the role of ALKBH5 in the pathogenesis of PD.

Quan et al. analyzed two datasets from GEO database: GSE120306 and GSE22491, and found that the m6A recognition protein HRNPC was significantly down-regulated in the PD group [44]. Subsequent research validated the protective effect of HRNPC overexpression on dopaminergic neurons in cell models. Since a portion of patients’ onset is due to pathogenic gene mutations associated with PD, Qin et al. used molecular inversion probes for targeted sequencing to investigate whether gene variants of ten m6A-related proteins were associated with sporadic PD in Han Chinese individuals [45]. They found no significant association between the two. However, their analysis has certain limitations, for example, the researchers did not adjust for genetic correlation of kinship in this study. The correlation between m6A RNA modification and PD requires larger and more racially diverse samples.

We concluded the current results related to m6A modification in PD clinical research. The overall level of m6A modification and the expression of certain m6A regulators show significant differences in the brains of PD patients. This suggests that m6A has the potential to become a biomarker for PD. However, research on m6A in PD patients is still limited. It remains unclear whether the modification levels of m6A in other brain regions and the expression levels of other m6A modification proteins differ from those in normal control groups. Advancing clinical research on m6A modification in PD patients is crucial for revealing the pathological mechanisms of PD and promoting the development of diagnosis and treatment.

## 3. Regulation of m6A RNA Modification in Dopaminergic Neurons

### 3.1. m6A RNA Modification Effect on Dopamine Metabolism

**Figure 2:**
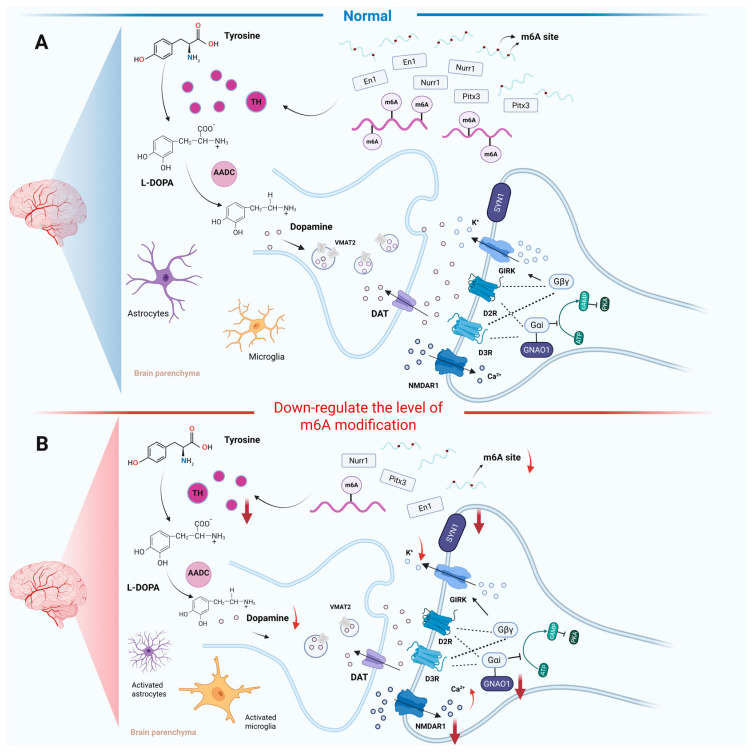
The effect of m6A RNA modification levels on dopaminergic metabolism and transmission. (**A**) The dopaminergic metabolism and transport system in normal condition. (**B**) In the condition of down-regulated m6A modification, the expression of TH-related transcription factors, including Nurr1, Pitx3, and En1, were decreased and subsequently inhibited TH expression, which further reduced dopamine synthesis and release. Down-regulated m6A modification also inhibited the D2R-D3R-GIRK pathway, which affected dopaminergic transmission. Created with BioRender.com.

In the study of the dopaminergic system and its associated neurological functions, the role of m6A modification has emerged as a significant area of research. Reducing m6A modification levels can affect transcription factors of essential enzyme in the dopamine metabolic pathway, thereby regulating dopamine synthesis. A hypomethylated environment can disrupt dopamine signaling pathways and the expression of relevant proteins (Figure 2). In PD, patients exhibit a significant reduction in dopamine levels within the SN [46].This dopaminergic neuron loss disrupts basal ganglia circuitry, leading to impaired motor function [47]. Tyrosine hydroxylase (TH) plays a crucial role in the synthesis of dopamine and catalyzes the conversion of the amino acid L-tyrosine to L-3,4-dihydroxyphenylalanine (L-DOPA) [48]. Subsequently, aromatic L-amino acid decarboxylase (AADC) converts L-DOPA into dopamine. Once synthesized, dopamine is actively transported into synaptic vesicles via the vesicular monoamine transporter 2 (VMAT2) for storage [49]. During neuronal stimulation, dopamine is released into the synaptic cleft, where it binds to pre- and postsynaptic receptors to mediate neurotransmission. Its action is terminated primarily through reuptake into presynaptic neurons via the dopamine transporter (DAT) and enzymatic degradation [50] (Figure 2A). Emerging evidence highlights the critical role of m6A modification in maintaining dopamine metabolism through the regulation of TH and associated transcriptional networks. Teng et al. found that the deletion of METTL14 in mouse SN led to a reduction in TH expression and the three transcription factors closely related to TH: nuclear receptor-related factor 1 (Nurr1), paired-like homeodomain transcription factor 3 (Pitx3), and engrailed-1 (En1) [51] (Figure 2B). This regulatory relationship was further corroborated in PD models. Yu et al. reported that 1-methy1-4-phenyl-1,2,3,6-tetrahydropyridine (MPTP)-induced PD mice exhibited marked TH reduction in both the SN and striatal regions, accompanied by distinct regional alterations in the m6A regulatory proteins [52]. The protein expressions of ALKBH5 and IGF2BP2 were up-regulated in the SN, while the readers, including YTHDF1 and fragile X messenger ribonucleoprotein 1 (FMR1), were down-regulated. For the striatum, FMR1 and the methyltransferase casitas B-lineage lymphoma-like 1 (CBLL1) were up-regulated, while IGF2BP3, METTL3 and RBM15 were down-regulated. Moreover, vitro finding by Geng et al. found that knocking down FTO effectively restored TH expression in the 1-methyl-4-phenylpyridinium (MPP+)-treated MN9D cells [38]. These collective findings suggest a potential mechanism whereby PD pathogenesis induces brain region-specific dysregulation of m6A, ultimately modifying m6A patterns that critically influence dopaminergic neuron metabolism through TH-mediated pathways.

Dopamine is stored in synaptic vesicles and released via calcium-dependent exocytosis, exerting its physiological effects through interaction with dopamine receptors. Dopamine receptors belong to a superfamily of G protein-coupled receptors, which are divided into two receptor families [53]: the D1-like family (D1 and D5) and the D2-like family (D2, D3 and D4), associated with Gα or Gi-mediated transduction systems, respectively, activating or inhibiting cyclic adenosine monophosphate-mediated signaling pathways. Emerging evidence suggests this signaling pathway may regulate dopamine homeostasis through synthesis, storage, and release mechanisms, potentially influencing PD progression [54]. Mechanistically, Hess et al. demonstrated that FTO downregulation disrupts the dopamine receptor type 2 and type 3-G protein-coupled inwardly rectifying potassium channel (D2R-D3R-GIRK) channel complex in midbrain dopaminergic neurons [55] (Figure 2B). Under physiological conditions, the activation of D2R and D3R promotes the opening of GIRK channels, leading to intracellular potassium ion efflux, which generates inhibitory signals and reduces excitability [56]. The knockout of FTO inhibits the opening of GIRK channels by targeting and regulating guanine nucleotide-binding protein O subunit α (GNAO1), N-methyl-D-aspartate receptor 1 (NMDAR1), and synaptic vesicle protein 1 (SYN1), thereby affecting the transmission of inhibitory neural signals. In the midbrain and striatum of FTO-deficient mice, global m6A modification of mRNAs was analyzed using methylated RNA immunoprecipitation sequencing (MeRIP-Seq) combined with next-generation sequencing. It was discovered that the adenosine methylation of mRNAs encoding proteins specifically associated with the dopamine signaling pathways, such as DRD3 and GNAO1, was increased. This finding reveals that FTO may regulate dopaminergic transmission through demethylation. We hypothesize that m6A dysregulation could potentially disturb neurotransmitter in dopaminergic circuits and modulate excitatory-inhibitory balance within midbrain signaling networks.

### 3.2. m6A RNA Modification Effect on the Survival of Dopaminergic Neurons

**Figure 3:**
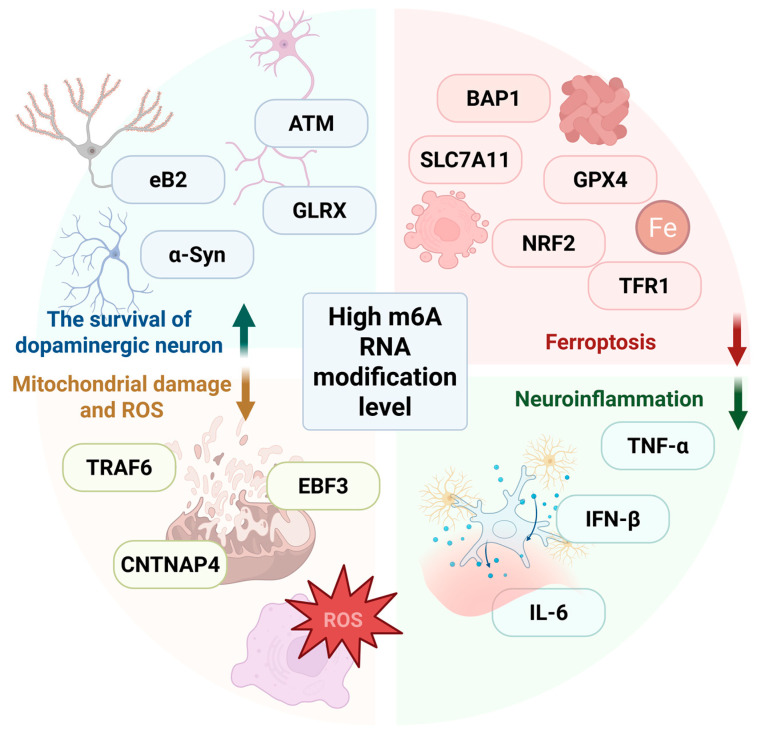
Regulation of m6A RNA modification in dopaminergic neurons survival. By knocking-down demethylase FTO or overexpressing methyltransferases like METTL14 and METTL3, the overall m6A level can be elevated, thereby establishing a hypermethylated environment. This environment typically exerts a neuroprotection effect on dopaminergic neurons. The underlying protective mechanisms involve enhancing the survival of dopaminergic neuron, reducing mitochondrial damage, ferroptosis, and neuroinflammation. These effects are achieved through the regulation of the expression of key related proteins by m6A RNA modification. Created with BioRender.com.

Dysregulation of m6A regulatory factors, that leads to high m6A RNA modification level, exerts significant effects on dopaminergic neuron homeostasis (Figure 3). In the study involving PC12 cells induced by the neurotoxin 6-hydroxydopamine (6-OHDA) and the striatum of rat models of PD, Chen et al. found that overexpression of FTO in dopaminergic cells or downregulating m6A levels using the m6A inhibitor cycloleucine led to an up-regulation of NMDAR1 expression [22]. These processes promoted calcium ion efflux, increased oxidative stress levels, and ultimately resulted in the apoptosis of dopaminergic neurons. This study provided the first evidence implicating FTO-mediated m6A demethylation in PD-associated dopaminergic degeneration. Subsequently, Selberg et al. developed a blood–brain barrier permeable small molecule inhibitor of FTO, which showed promise in promoting the survival of midbrain dopaminergic neurons [57]. This inhibitor exhibited superior neuroprotective efficacy and pharmacokinetic properties compared to the team’s previously reported ALKBH5-targeted compound [53]. Geng et al. demonstrated that FTO might exert a regulatory effect on dopaminergic neurons through ataxia telangiectasia mutated (ATM). The delivery of siFTO using exosomes derived from mesenchymal stem cells inhibited the expression of ATM mRNA in a highly methylated environment [38]. Given ATM regulates the DNA damage response, low expression of ATM can alleviate the apoptosis of dopaminergic neurons in PD. Early B Cell Factor 3 (EBF3), a member of the highly evolutionarily conserved EBF-transcription factor family, is involved in neuronal development [58]. siFTO up-regulates m6A methylation of *EBF3*, extending the half-life of *EBF3* mRNA [59]. *EBF3* attenuates PD by activating CNTNAP4 (a protein associated with neuronal differentiation) transcription. Functionally, in vivo and in vitro experiments demonstrate that high methylation levels can overexpress *EBF3*, promote CNTNAP4 transcription expression, ameliorate PD behavioral deficits, and inhibit dopamine neuron apoptosis. Nuclear factor erythroid 2-related factor-2 (NRF2) can regulate genes related to inflammatory responses, iron management, metabolic pathways, mitochondrial bioenergetics, and protein homeostasis [60]. Pang et al. have shown that FTO removes m6A modifications on *NRF2* mRNA, thereby impairing *NRF2* mRNA stability and exacerbating ferroptosis in MPP+-treated SH-SY5Y neuronal cells [61]. Furthermore, another study validated that m6A RNA modification affects ferroptosis in dopamine neurons in a PD model through another pathway, both in vivo and in vitro. The m6A-binding protein YTHDF2 recognizes and degrades the methylated mRNA of *BAP1*, leading to reduced BAP1 stability. BAP1 cooperates with *p53* to decrease the expression level of solute carrier family 7 member 11 (SLC7A11). A hypermethylated environment inhibits BAP1 and up-regulates SLC7A11, ultimately leading to the suppression of MPP+-induced ferroptosis [62].

However, the pathophysiological role of FTO appears context-dependent in metal-induced PD models. In the cell and animal PD models induced by arsenite, arsenic exposure remarkably increased the m6A modification levels, while FTO was found to alleviate deficits in dopaminergic neurotransmission caused by arsenite [63]. Another study indicated that the Foxo3a/FTO/m6A/ephrin-B2 (*eB2*)/YTHDF2 pathway plays a crucial role in manganese (Mn)-induced PD models [64]. Mn induces the up-regulation of Foxo3a, which in turn down-regulates FTO expression, leading to an increase in the m6A modification level of *eB2*. After being recognized by YTHDF2, *eB2* mRNA is degraded, ultimately resulting in damage to dopaminergic neurons in the SN-striatum projection system and causing motor dysfunction in animals. In this pathway, high m6A levels achieved by regulating the expression of FTO does not exert neuroprotective effects as it does in other pathways, overexpression of FTO can reverse the damage to dopaminergic neurons in PD. In summary, FTO as an m6A regulatory factor, affects the survival of dopaminergic neurons in animal and cell models of PD. The discovery of the mechanism of FTO highlights the complexity of m6A in regulating the survival of dopamine neurons and provides a new avenue for the treatment of PD.

Current evidence manifests that m6A may influence the survival of dopaminergic neurons through multifaceted mechanisms, such as affecting the accumulation of α-Syn, inflammatory processes, or mitochondrial function. It is well known that the aggregation of α-Syn is essential for the pathophysiological features of PD [65]. The presence of Lewy bodies in SN leads to disruptions in the autophagy-lysosome system, mitochondrial dysfunction, and endoplasmic reticulum stress, ultimately resulting in damage to dopaminergic neurons [31]. Xiao et al. demonstrated that METTL14 overexpression suppresses α-Syn aggregation by enhancing m6A deposition on α-Syn transcripts, thereby facilitating YTHDF2-dependent mRNA degradation [36]. Furthermore, Zhang et al. have confirmed that METTL14 can reduce the expression of tumor necrosis factor receptor-associated factor 6 (TRAF6) through m6A modification [66]. The activation of the cyclic GMP-AMP synthase (cGAS)-stimulator of interferon genes (STING) pathway is inhibited, which in turn decreases ferroptosis, improves mitochondrial function and suppresses reactive oxygen species (ROS) production in PD. In the previous text, Quan et al. found that the expression of HNRNPC is down-regulated in the PD group [44]. The functional assays show that HNRNPC overexpression suppresses pro-inflammatory mediators, including interferon-β (IFN-β), interleukin-6 (IL-6), and tumor necrosis factor-alpha (TNF-α), while enhancing dopaminergic neuron viability. Elevated IL-6 and TNF-α levels are mechanistically linked to PD progression via microglial activation and synaptic dysfunction [67]. Glutaredoxin (GLRX) has been found to regulate the death of dopaminergic neurons and promotes cytokine production in microglial cells [68]. Nuclear factor erythroid 2-like 1 (NRF1) can bind to the promoter of *METTL3 *t o increase its expression and enhance the m6A modification of *GLRX* mRNA in MPTP-induced mice, ultimately inhibiting motor function impairment and degeneracy of dopaminergic neurons [69]. Moreover, Nurr1 can recruit the CoREST corepressor complex, preventing the occurrence of neuroinflammation and reducing the loss of dopaminergic neurons by limiting the production of neurotoxic mediators from microglia and astrocytes [70]. METTL14 can affect the expression of Nurr1 [51], indicating that there may be a potential mechanism by which m6A modification influences the formation of the Nurr1-CoREST complex, thereby inhibiting the inflammatory cascade and protecting dopaminergic neurons. In summary, it can be indicated that an environment with high levels of m6A modification can exert protective effects on dopaminergic neurons through various pathways.

m6A is involved in the multi-level regulation of dopamine neurons. Artificial manipulation of m6A modification levels is often accompanied by changes in the activity of various signaling pathways or other biological processes. Meanwhile, m6A can precisely regulate the expression and function of proteins related to dopamine synthesis, transport, and release, ultimately affecting dopamine metabolism and neuronal activity. This provides a theoretical basis for a deeper understanding of the pathogenesis of PD and offers potential therapeutic strategies. However, the protective role of m6A in dopamine neurons requires further research. It is crucial to clarify which m6A regulatory factors lead to high levels of m6A modification that can protect dopamine neurons, as well as which PD models exhibit this protective effect. This understanding will establish whether m6A can serve as a therapeutic target for different pathogenic factors in PD.

## 4. m6A RNA Modification of PD Pathogenic Genes

m6A modification at the post-transcriptional level determines the fate of mRNA molecules, affecting nearly all important biological processes and being implicated in various diseases [71]. Detection methods for m6A can be broadly categorized into three types: global level of m6A detection, single-gene m6A site detection, and high-throughput m6A gene sequencing technology [72]. For total m6A levels detection, RNA immunoblotting methods, such as dot-blot, offer stronger operability and save time. However, dot-blot cannot accurately quantify and determine the exact locations of m6A sites, making it primarily useful for comparing overall m6A levels between different groups [73]. Liquid chromatography-tandem mass spectrometry (LC-MS) provides high sensitivity and confidence for the identification and quantification of modified RNA, allowing for the most precise determination of overall m6A modification levels under different experimental conditions and in various cell types [74]. MeRIP-Seq or m6A-Seq is the first high-throughput method that locates m6A in a whole transcriptome manner [75]. This method fragments mRNA, enriches mRNA fragments using m6A antibodies, and prepares libraries for next-generation sequencing. MeRIP-Seq has been widely used to reveal the dynamics of m6A modification and downstream genes in different physiological or pathological processes, such as embryonic development and cancer progression. However, MeRIP-Seq cannot accurately pinpoint the exact locations of m6A sites. This limitation has been addressed by m6A individual-nucleotide resolution cross-linking and immunoprecipitation (miCLIP) or m6A-CLIP, which uses UV to cross-link RNA-antibody complexes directly [76], purifying and preparing iCLIP libraries. m6A-CLIP/miCLIP can reveal m6A maps with single-base resolution. Subsequently, the m6A-REF-seq/MAZTER-seq technology was developed, which not only has single-base resolution but also does not rely on antibodies, offering high specificity [77].

The development of m6A detection methods has greatly facilitated research on the functions and mechanisms of m6A, generating vast amounts of data and leading to the emergence of m6A-related databases that provide data support for researchers. M6A2Target (http://m6a2target.canceromics.org) primarily focuses on identifying downstream target genes of m6A-related “writer”, “eraser”, and “reader” proteins (WERs) [78]. In the field of m6A modification research, understanding the dynamic modifications of WERs on target genes is crucial for exploring the dynamic reversibility of m6A modifications, and the M6A2Target database provides key support for this. RMVar (http://rmvar.renlab.org) allows for the querying of m6A modification sites in different genes, as well as closely related variant information, covering the intrinsic connections between these related variants and various diseases [79]. The RMBase database (http://bioinformaticsscience.cn/rmbase/, accessed on 15 April 2025), constructed by a team from Sun Yat-Sen University, has been upgraded to version 3.0 since its establishment in 2015, and can be used to explore transcriptomic maps, biological occurrences, interactions, and functions of various RNA modifications across different species [80]. m6A-Atlas (www.xjtlu.edu.cn/biologicalsciences/atlas, accessed on 15 April 2025) is a comprehensive database that reveals the m6A transcriptome modification status and can be used to query m6A modification sites, RNA-binding proteins (RBPs), and miRNA regulation information [81]. Additionally, there is also a dedicated website for predicting m6A sites on RNA sequences of different genes in various mammals—SRAMP [82], which was developed in 2016 and has since been widely used by researchers and offers a simple prediction method and diverse prediction models. Recently, the website was upgraded to SRAMP2 (www.cuilab.cn/deepsramp, accessed on 15 April 2025), which performs better than its predecessor and shows improved accuracy. These databases each have their own focus, aiding researchers in selecting suitable data sources based on their needs, while assessing genetic variations in RNA modifications provides new perspectives for understanding the mechanisms of diseases.

Approximately 5–10% of PD can be attributed to genetic variations in PD-related pathogenic genes, while other causes are associated with complex genetic susceptibility and environmental factors [83]. Studying the potential genetic basis of PD is crucial for understanding its pathogenesis. In the process of exploring the regulatory role of m6A in PD, the specific biological functions of m6A in different PD-related genes are significant. We selected the RMBase database and SRAMP for a more comprehensive prediction and screening of m6A site information. Firstly, we used SRAMP to predict medium to high confidence m6A sites for four PD-related genes derived from humans and mice: *SNCA*, *PRKN*, *PINK1*, and *LRRK2* [84], and checked whether these sites exist in the RMBase database. The sites with higher confidence and supported by the database were filtered out to provide a foundation for further research on the biological functions of m6A in PD-related genes (Table 1). The m6A modification sites of *SNCA* were primarily obtained through studying the roles of various m6A-related proteins in cancer, stem cells, and the nervous system. METTL14 can regulate the expression and aggregation of α-Syn by affecting the m6A modification level at position 615 in the CDS region of α-Syn, which will have an impact on the process of PD [36]. Aside from *SNCA*, there are no individual articles reporting the specific functions of m6A loci for the other three PD-related genes or their relevance to PD; however, we speculate that their m6A modification levels similarly influence PD. Based on the information regarding the m6A sites of these four genes shown in the databases, it can be summarized with these following conclusions: in terms of species, humans have more m6A modification sites than mice. Methyltransferase-targeted mutagenesis studies and in vitro analysis suggest that m6A appears to be limited to adenosine within the sequence motif DRACH , primarily located near gene terminators, 3′ untranslated regions (3′-UTR), and internal long exons, as confirmed by the information in the table. The main experimental methods summarized in the database for detecting m6A are MeRIP-Seq and m6A-Seq. While this review focuses on four genes here, other key genes, such as *GAB1* and *PARK7*, also have numerous m6A modification sites. Moreover, we found that SRAMP can predict the m6A modification sites in rats. However, there is a lack of relevant information about rats in the integrated database, indicating a deficiency of datasets concerning rats. Research on the association between m6A modifications in individual genes and PD is relatively lacking and requires further exploration. The functionality of the existing m6A modification sites identified by SRAMP also remains to be determined.

## 5. Discussion

Recent studies have increasingly emphasized the crucial role of m6A RNA modification in the pathogenesis of neurological diseases, affecting their occurrence and progression. PD is a common neurodegenerative disease caused by the death of dopaminergic neurons in the SN of midbrain. This review summarized the recent progress of m6A RNA modification in PD clinical research, discussed the effects of m6A on dopaminergic cells from various perspectives and made reasonable speculations about other possible pathways, and outlined the m6A modification sites of specific pathogenic genes (*SNCA*, *PRKN*, *PINK1*, and *LRRK2*) associated with PD. So far, clinical sample results provide direct evidence for the association between m6A modification and PD. The occurrence and progression of PD are accompanied by alterations in m6A modification levels and the expression of m6A-related proteins. In cellular or animal models of PD, high levels of m6A modification can protect dopaminergic neurons from apoptosis under certain conditions. Thus, it is reasonable to infer that the levels of m6A modification or related regulatory proteins could serve as biomarkers for diagnosis and therapeutic targets in PD.

However, despite progress in understanding the regulatory effects of m6A RNA modification on PD, several limitations persist. In clinical research, the universality and representativeness of changes in m6A modification in the blood or brain tissues of PD patients need to be verified. It remains unclear whether m6A modification levels and certain untested m6A-related proteins differ across various brain regions in PD patients compared to normal controls. The correlation between the degree of reduction in m6A modification levels and PD severity requires further exploration. Additionally, current detection techniques for m6A have shortcomings, making it challenging to establish them as reliable biomarkers. While antibody-based immunoprecipitation sequencing can identify m6A modification sites, variations in antibody specificity and affinity can lead to false-positive or false-negative results, lacking precise detection at single-base resolution. Although third-generation sequencing technologies can provide single-base level information, issues such as high costs and low throughput hinder large-scale sample testing, limiting comprehensive analysis of m6A modification in PD samples. This limits a comprehensive and accurate analysis of m6A modification in PD clinical samples, affecting our understanding of the role of m6A in the disease.

While the regulatory roles of m6A modification in dopamine metabolism, signaling pathways, neuronal apoptosis, oxidative stress, ferroptosis and inflammatory responses have been identified, existing studies are not yet comprehensive. The precise regulation of gene expression in dopaminergic neurons by m6A modification remains unclear. The dynamic changes in m6A across different cell types and stages of PD need further confirmation. Furthermore, research on the interactions of m6A modification with other cellular processes also requires improvement. The specific roles and regulatory networks of m6A in conjunction with autophagy, mitochondrial function, and other processes in PD are still not well understood. There is a notable interaction between m6A modification and autophagy. For instance, METTL3 mediates m6A RNA modification that inhibits autophagy [85], while YTHDF2 promotes autophagy [86]. The suppression or up-regulation of autophagy by m6A modification may depend on the level of m6A methylation or the effects of downstream targets [87]. However, specific studies on the role of m6A in autophagy in PD are lacking, which limits our overall understanding of m6A in PD pathogenesis, lmiting the overall understanding of m6A in the pathogenesis of PD. Recently, some progress has been made regarding the role of m6A modification in regulating ferroptosis of dopaminergic neurons in PD, but its molecular mechanisms require further investigation. Future research could focus on elucidating the spatiotemporal-specific roles of specific m6A proteins, such as the YTHDF family or eraser enzymes, in neuronal iron homeostasis and lipid peroxidation. Moreover, it is crucial to clarify the causal relationship map between m6A modification and ferroptosis signaling, and whether its regulatory hierarchy is affected by α-syn pathology or the neuroinflammatory microenvironment. Mechanism studies suggest that regulating the expression or activity of m6A modification-related enzymes in cellular and animal models can improve the pathological features associated with PD. Nonetheless, translating these research findings into clinical treatment methods still faces significant challenges.

In the future, there are several promising research directions regarding m6A RNA modification in PD that are still worthy of in-depth exploration. A comprehensive understanding of the mechanisms of m6A modification in PD is essential. Identifying small molecules or biological agents that can specifically regulate the activity of m6A modification-related enzymes and developing novel therapeutic strategies are crucial. Progress has been made in developing small molecule inhibitors targeting FTO, with some inhibitors for anti-inflammatory or anti-tumor purposes already established [88]. In 2021, Selberg et al. developed small molecule compounds that suppress FTO to protect dopaminergic neurons, laying the groundwork for optimizing and clinically applying FTO inhibitors for PD treatment [57]. Additionally, utilizing gene therapy techniques to introduce or knock out specific m6A modification-related genes could achieve regulation of m6A modification levels, potentially playing a significant role in the clinical treatment of PD in the future. In summary, research on the regulatory effects of m6A on PD is vital for deepening the understanding of the disease, developing novel therapeutic methods, and identifying early biomarkers.

## 6. Conclusions

m6A RNA modification, an epigenetic modification that is widely present on mRNA and non-coding RNA, has a significant impact on the development of the nervous system, as well as the occurrence and progression of PD. Compared with healthy controls, the overall level of m6A modification is significantly reduced in PD patients. Notability, there are vital alterations in the expression of specific m6A-associated proteins, further highlighting the pivotal role of m6A modification in the pathophysiology of PD. Existing research indicates that changes in m6A modification caused by altered expression of m6A-related proteins can affect key enzymes expression and signaling pathways involved in dopamine metabolism. Intriguingly, most reports showed that high levels of m6A modification can protect the survival of dopaminergic neurons through various pathways, including improving mitochondrial function, reducing oxidative stress, neuroinflammation or ferroptosis. Furthermore, at the single-gene level, the impact of m6A modification on the functions of PD pathogenic genes is deserving our focused attention. Presently, there are several specialized m6A-related databases and online prediction websites that serve as valuable resources to advance further research. We specifically selected four important pathogenic genes associated with PD (*SNCA*, *PRKN*, *PINK1*, and *LRRK2*), and identified that m6A sites do exist on mRNA of these genes with higher reliability according by the databases, laying a foundation for further investigation into the biological functions of m6A modification in PD pathogenic genes. 

However, the study of m6A RNA modification in PD is still in the initial state, and many specific areas deserve further development. The regional variability and representativeness of total m6A levels and the associated proteins in PD patients remain to be confirmed. The precise regulation of dopaminergic neurons under different PD models by m6A modifications is still unclear. The potential therapy of m6A demethylase-related inhibitors in PD is worth exploitation. The progress of these studies is crucial for revealing the role of m6A RNA modification in the pathogenic mechanisms of PD. Moreover, it offers novel perspectives and strategic directions for the diagnosis and treatment of PD.

## Figures and Tables

**Table 1:** The potential m6A sites on mRNA of *SNCA*, *PRKN*, *PINK1*, and *LRRK2*.

Gene	Mod ID	Predictive Reliability	Technique	Region	Data Source
*SNCA Homo*	m6A_site_644495	High confidence	MeRIP-seq	CDS	GSE48037GSE76414
*SNCA Homo*	m6A_site_644502	High confidence	MeRIP-seq	CDS	GSE107954GSE93911
*SNCA Homo*	m6A_site_644494	Moderate confidence	MAZTER-Seq	CDS/Intron	GSE129842
*SNCA Mus*	m6A_site_505923	High confidence	MeRIP-seq	CDS	GSE47215
*SNCA Mus*	m6A_site_505932	Moderate confidence	MeRIP-seq	Exon	GSE100528GSE29714
*PRKN Homo*	m6A_site_742128	Very High confidence	MeRIP-seq	UTR	GSE93911GSE29714
*PRKN Homo*	m6A_site_742132	Very High confidence	MeRIP-seq	UTR	GSE120229GSE29714
*PRKN Homo*	m6A_site_742134	Very High confidence	m6A-seq	Exon/Intron	GSE93911
*PRKN Homo*	m6A_site_742126	High confidence	m6A-seq	UTR	GSE93911GSE87515
*PRKN Homo*	m6A_site_742129	High confidence	MeRIP-seq/m6A-seq	UTR	GSM3396437GSM928399
*PRKN Homo*	m6A_site_742131	High confidence	MeRIP-seq/m6A-seq	UTR	GSE120229GSE29714
*PRKN Homo*	m6A_site_742135	High confidence	m6A-seq	Exon/Intron	GSE93911
*PRKN Homo*	m6A_site_742136	High confidence	m6A-seq	Exon/Intron	GSE93911
*PRKN Homo*	m6A_site_742137	High confidence	m6A-seq	Exon/Intron	GSE93911
*PRKN Homo*	m6A_site_742133	Moderate confidence	MeRIP-seq	UTR	GSE120229GSE29714
*PRKN Mus*	m6A_site_250551	Very High confidence	MeRIP-seq	Exon/Intron	GSE47215GSE100528
*PRKN Mus*	m6A_site_250552	Very High confidence	MeRIP-seq	Exon/Intron	GSE47215GSE100528
*PRKN Mus*	m6A_site_250547	High confidence	MeRIP-seq	Exon/Intron	GSE47215GSE100528
*PRKN Mus*	m6A_site_250550	High confidence	MeRIP-seq	Exon/Intron	GSE47215GSE100528
*PRKN Mus*	m6A_site_250553	High confidence	MeRIP-seq	Exon/Intron	GSE47215GSE100528
*PRKN Mus*	m6A_site_250556	High confidence	MeRIP-seq	Exon/Intron	GSE47216
*PRKN Mus*	m6A_site_250546	Moderate confidence	MeRIP-seq	Exon/Intron	GSE100528
*PINK1 Homo*	m6A_site_12074	Very High confidence	MeRIP-seq/m6A-seq	Exon	GSE102493 GSE110320
*PINK1 Homo*	m6A_site_12076	Very High confidence	MeRIP-seq/m6A-seq	Exon	GSE102493 GSE110320
*PINK1 Homo*	m6A_site_12028	High confidence	m6A-seq	Exon	GSE102493 GSE46705
*PINK1 Homo*	m6A_site_12013	Moderate confidence	m6A-seq	CDS	GSE102493GSE125780
*PINK1 Homo*	m6A_site_12019	Moderate confidence	m6A-seq	Exon	GSE87190GSE93911
*PINK1 Homo*	m6A_site_12032	Moderate confidence	MeRIP-seq/m6A-seq	Exon	GSE102493GSE110320
*PINK1 Homo*	m6A_site_12055	Moderate confidence	m6A-seq	Exon	GSE110320GSE87190
*PINK1 Mus*	m6A_site_440098	Very High confidence	MeRIP-seq/m6A-seq	CDS	GSE47215GSE53244
*PINK1 Mus*	m6A_site_440091	High confidence	MeRIP-seq/m6A-seq	CDS	GSE47215 GSE98085
*PINK1 Mus*	m6A_site_440082	Moderate confidence	MeRIP-seq/m6A-seq	UTR	GSE100528GSE47215
*PINK1 Mus*	m6A_site_440095	Moderate confidence	m6A-seq	Exon	GSE98085
*LRRK2 Homo*	m6A_site_182822	Very High confidence	m6A-CLIP	CDS	GSE71154
*LRRK2 Mus*	m6A_site_220644	Very High confidence	m6A-seq	CDS	GSE98085
*LRRK2 Mus*	m6A_site_220649	Very High confidence	MeRIP-seq	Exon	GSE47215
*LRRK2 Mus*	m6A_site_220651	Very High confidence	MeRIP-seq	Exon	GSE100528
*LRRK2 Mus*	m6A_site_220652	Very High confidence	MeRIP-seq	Exon	GSE100528
*LRRK2 Mus*	m6A_site_220648	Very High confidence	MeRIP-seq	Exon	GSE47215
*LRRK2 Mus*	m6A_site_220642	Moderate confidence	MeRIP-seq/m6A-seq	CDS	GSE115106GSE61995

## Abbreviations

The following abbreviations are used in this manuscript:

ALKBH5	AlkB homologue 5 protein
ATM	Ataxia telangiectasia mutated
En1	Engrailed-1
FTO	Fat mass and obesity associated proteins
GNAO1	Guanine nucleotide-binding protein O subunit α
HNRNPC	Heterogeneous nuclear ribonucleoprotein C
IFN-β	Interferon-β
IL-6	Interleukin-6
LC-MS	Liquid chromatography-tandem mass spectrometry
MeRIP-Seq	Methylated RNA immunoprecipitation sequencing
Mn	Manganese
MPTP	1-methy1-4-phenyl-1,2,3,6-tetrahydropyridine
MPP+	1-methyl-4-phenylpyridinium
NMDAR1	N-methyl-D-aspartate receptor 1
Nurr1	Nuclear receptor-related factor 1
PBMCs	Peripheral blood mononuclear cells
PD	Parkinson’s disease
Pitx3	Paired-like homeodomain transcription factor 3
ROS	Reactive oxygen species
SN	Substantia nigra
SYN1	Synaptic vesicle protein 1
SLC7A11	solute carrier family 7 member 11
TH	Tyrosine hydroxylase
TRAF6	Tumor necrosis factor receptor-associated factor 6
TNF-α	Tumor necrosis factor-alpha
α-Syn	α-synuclein
m6A	*N*6-methyladenosine
m6A-SNPs	m6A-related single nucleotide polymorphisms
RBPs	RNA-binding proteins
